# Single‐cell transcriptomics reveals critical role of CD74 in bone marrow–derived mesenchymal stem cells

**DOI:** 10.1093/stcltm/szag082

**Published:** 2026-09-18

**Authors:** Qinqin Xu, Carson Curtis, Jiahao Zhang, Arjun Verma, Aaron J Sheppard, Jeffrey Deng, Hicham Drissi, Shane Barton, Patrick Massey, Yufeng Dong

**Affiliations:** Department of Orthopedic Surgery, LSU Health-Shreveport, Shreveport, LA 71130, United States; Department of Neurology, LSU Health-Shreveport, Shreveport, LA 71130, United States; Department of Orthopedic Surgery, LSU Health-Shreveport, Shreveport, LA 71130, United States; Department of Neurology, LSU Health-Shreveport, Shreveport, LA 71130, United States; Department of Orthopedic Surgery, LSU Health-Shreveport, Shreveport, LA 71130, United States; Department of Orthopedic Surgery, LSU Health-Shreveport, Shreveport, LA 71130, United States; Dartmouth Geisel School of Medicine, Dartmouth College, Hanover, NH 03755, United States; Department of Orthopedic Surgery, Emory University School of Medicine, Atlanta, GA 30329, United States; Department of Orthopedic Surgery, LSU Health-Shreveport, Shreveport, LA 71130, United States; Department of Orthopedic Surgery, LSU Health-Shreveport, Shreveport, LA 71130, United States; Department of Orthopedic Surgery, LSU Health-Shreveport, Shreveport, LA 71130, United States

**Keywords:** bone marrow–derived mesenchymal stem/stromal cells, single-cell RNAseq, osteogenesis, CD74

## Abstract

**Background:**

Human bone marrow aspirates have been widely used for skeletal tissue regeneration. However, clinical outcomes of the tissue repair after transplantation are variable.

**Methods:**

In this study, we analyzed the molecular signatures and osteogenic differentiation potential of bone marrow-derived mesenchymal stromal cells (BMSCs) from different donors.

**Results:**

Our results clearly showed that BMSCs isolated from different patients exhibited different osteogenic capacities and growth rates. Moreover, single-cell RNA sequencing revealed that these BMSCs expressed elevated levels of genes associated with early osteogenesis, chondrogenesis, and adipogenesis. Differentially expressed genes related to cell cycles were enriched in clusters from all 5 different samples, suggesting these genes play an important role in BMSC maintenance and differentiation. More importantly, the BMSCs with the lowest osteogenic differentiation potential showed a dramatic decrease in the expression of cell surface protein CD74 when compared to other osteogenic genes. Knockdown of CD74 in BMSCs with high osteogenic potential significantly reduced their osteogenic capacity.

**Conclusions:**

These findings suggest that CD74 is a novel predictive marker of outcomes following BMSC transplantation. Furthermore, CD74-high BMSCs may represent a subpopulation of bone marrow cells responsible for bone marrow aspirate-mediated tissue regeneration.

## Graphical Abstract

**Graphical Abstract:**
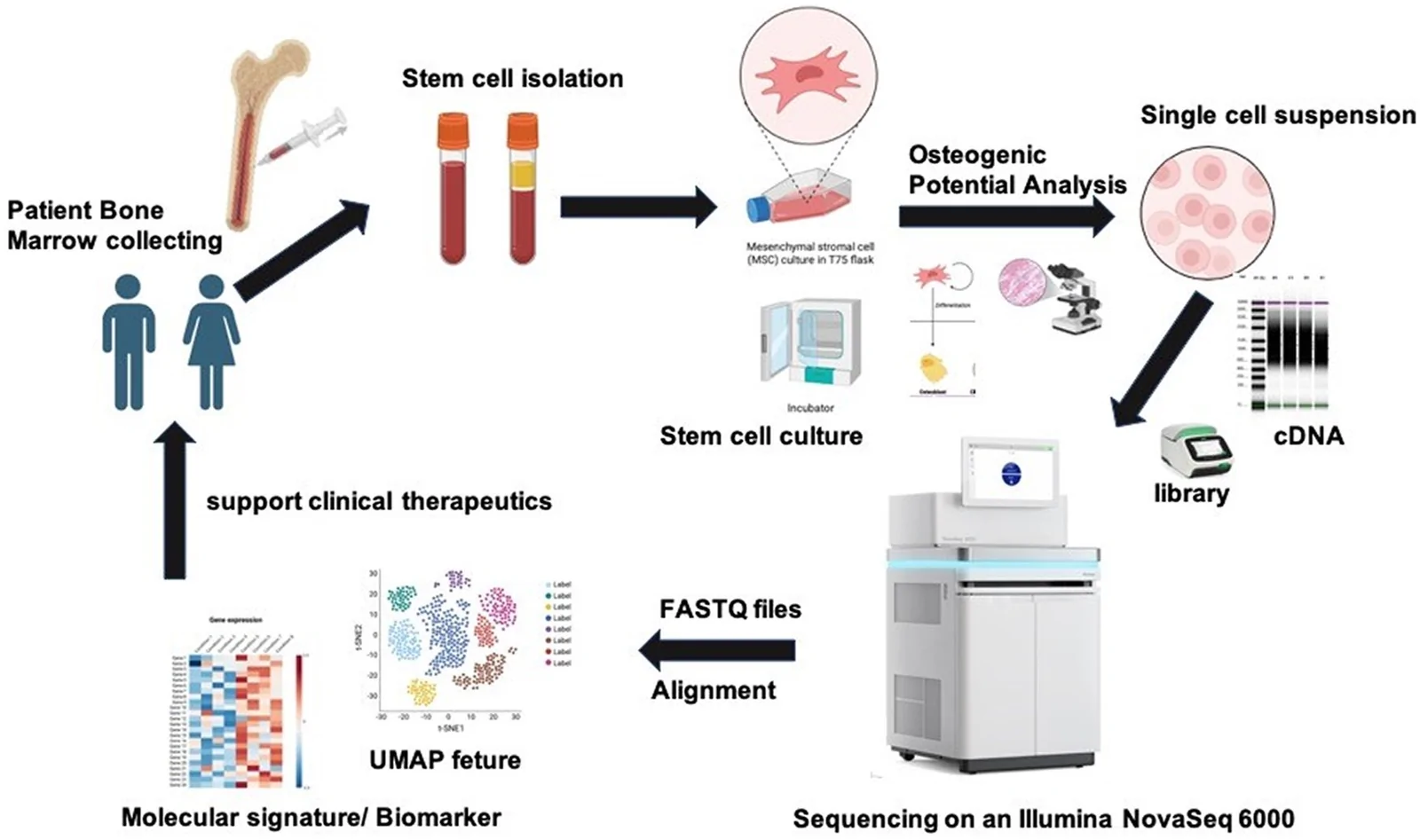

Significance statement This study identifies CD74 as a key predictive biomarker of osteogenic potential in human bone marrow–derived BMSCs. Through single-cell transcriptomic analysis and functional validation, we demonstrate that reduced CD74 expression is associated with diminished osteogenic capacity, while its knockdown directly impairs differentiation. These findings provide new insight into the molecular heterogeneity of BMSCs and highlight CD74 as a promising marker for selecting more functional stem cell populations. This work advances the development of more reliable, targeted strategies for bone regeneration and improves the therapeutic efficacy of bone marrow-based cell therapies.

## Introduction

Adult mesenchymal stem cells (MSCs) have been widely used for tissue engineering and repair due to their ability to self-renew and differentiate into specialized cell types. Based on their source, MSCs can be classified into bone marrow MSCs (BMSCs), adipose-derived MSCs (ASCs), perivascular stem cells (PSCs), induced pluripotent stem cell–derived MSCs (iPSC-MCSs), and genetically modified MSCs.^1^^,^^2^ Since BMSCs are relatively easy to harvest and exhibit strong differentiation potential into musculoskeletal tissues, including bone, cartilage, muscle, and adipose,^3^^,^^4^ these cells have been extensively studied for skeletal tissue regeneration. In a prospective case-controlled clinical study, a BMSC-soaked matrix not only had a comparable efficacy to posterolateral spinal fusion compared with traditional iliac crest autograft but also significantly reduced the risk of donor-site pain/neuroma.^5^ Another comparative study showed that patients with bone nonunion who received ex vivo-expanded autologous BMSCs by injection achieved successful union in 1 year, which is comparable to patients who received iliac crest autograft transplantation. Moreover, patients treated with BMSCs showed faster functional and radiographic improvement.^6^ Although these results are promising, the ability of BMSC to directly regenerate skeletal tissue is still under debate. For example, a review of 16 previously published studies and 807 patients did not find a significant difference between intra-articular injection of MSCs and placebo controls with chronic osteoarthritis,^7^ suggesting MSC injection provides little to no improvement in pain or physical function. Since BMSCs derived from different donors exhibit substantial variability in osteogenic potential, even when isolated under similar conditions,^4^^,^^8–10^ further studies are needed to identify the molecular mechanisms underlying MSC subpopulations that contribute to enhanced chondro-osteogenesis.

Recent advances in cell biology such as single-cell RNA sequencing provide powerful tools to investigate this variability, uncovering molecular signatures that can distinguish better-performing BMSCs. Here, we hypothesize that BMSCs from different patients exhibit distinct cellular compositions, transcriptomes, and cell–cell interactions. We aim to identify novel factors that drive enhanced chondro-osteogenic potential in BMSCs by integrating single-cell sequencing and gene knockout approaches. More importantly, elucidating BMSC cellular composition and downstream gene activation will allow us to identify useful markers for predicting clinical outcomes following BMSC transplantation.

## Methods

### Isolation of human bone marrow–derived stromal cells

Bone marrow samples were obtained from donors registered at the Department of Orthopedic Surgery, LSU Health Shreveport. Patient information and samples were collected under approval of the Louisiana State University Health Sciences Center Institutional Review Board (IRB ID MOD00004448_STUDY00000552). The human bone marrow–derived MSCs were isolated based on previous reports with modifications.^11^^,^^12^ Briefly, the collected bone marrow samples were adjusted to 10 mL with 1× phosphate-buffered saline (PBS) and transferred to a 50 mL conical tube through a 40-μm cell strainer. A 10× red blood cell lysis buffer was added, and the tube was gently vortexed immediately after addition. Samples were incubated at room temperature for 10-15 min while protected from light and then centrifuged at 300*g* for 10 min at room temperature. The supernatant was aspirated completely, and the cell pellet was washed by adjusting the volume to 45 mL with 1× PBS, followed by centrifugation at 400 *g* for 10 min at room temperature. The supernatant was then aspirated. The final pellet was resuspended in 10 mL cell growth media. Cells were plated at a density of approximately 0.5-1 million total per 10-cm culture dish in 10 mL culture medium. Cells were then cultured in phenol red-free α-MEM supplemented with 10% Fetal Bovine Serum–Heat Inactivated (FBS-HI) and antibiotics (100 U/mL penicillin and 100 μg/mL streptomycin, Invitrogen) at 37 °C in a humidified atmosphere containing 5% CO_2_.

### Determination of cell growth rate

Cell growth rate was measured by cell counting in a hemocytometer and WST-8/CCK8 assay (ab228554, Abcam).^13^ Cells were plated 5 × 10^3^ cells per well in 96-well plates or 5 000-20  000 cells per well in 24-well plates on day 0, and the medium was changed every 2 days until harvest for counting or performance of the WST-8/CCK8 assay. Cell counting using a hemocytometer was performed as described.^14^ Briefly, cells were harvested, and 10 μL of the cell suspension was loaded onto the hemocytometer. The chamber was placed under an inverted microscope using a 10x objective. Cells were counted in 4 of the “4X4” grid squares, averaged, and multiplied by 10^4^ to estimate the number of cells per mL. Duplicate samples were prepared, and the mean value was calculated. The WST/CCK8 assay was performed according to the manufacturer’s instructions. Briefly, 10 μL of WST-8 solution was added to each well of a 96-well plate (100 µL/well). Plates were incubated at 37 °C, 5% CO_2_ for 2 h, and absorbance was measured at 460 nm using a plate reader (BioTek Synergy LX multi-mode reader). Absorbance values were proportional to cell number.

### Cell surface staining for flow cytometry

Single-cell suspensions in Cell Staining Buffer (BioLegend #420201) were centrifuged at 350 *g* for 5 min. Viable cells were counted and resuspended in Cell Staining Buffer at a concentration of 5-10 × 10^6^ cells/mL, and 100 µL/tube of cell suspension (5-10 × 10^5^ cells/tube) was distributed. Antibodies were added at concentrations optimized by prior titration, and samples were incubated on ice for 15-20 min in the dark. Cells were washed twice with at least 2 mL of Cell Staining Buffer by centrifugation at 350× *g* for 5 min. The cell pellet was resuspended in 0.5 mL of Cell Staining Buffer, and 5 µL (0.25 µg/million cells) of 7-AAD Viability Staining Solution (BioLegend #420403) was added to exclude dead cells. After incubation on ice for 3-5 min in the dark, samples were analyzed by flow cytometry. A NovoCyte Quanteon flow cytometer was used to analyze surface marker expression of MSCs, including CD34 (Biolegend #343607), CD45 (Thermo Fisher Scientific #11-0451-81), CD73 (CiteAb #130-095-183), and CD105 (Abcam #ab11415-100), according to the criteria defined by the International Society for Cellular Therapy (ISCT).^15^

### Alkaline phosphatase staining

Cells were seeded into 12-well plates and allowed to adhere for 24 h before being cultured in osteogenic differentiation medium, with medium changed every other day. Cells were washed with PBS and fixed on days 10-14. Alkaline phosphatase (ALP) staining was performed^16^ using 1-Step NBT/BCIP (Thermo Fisher Scientific #34042) according to the manufacturer’s protocol.

### Alizarin Red S staining

For detection of calcification during osteogenic differentiation, cells were washed with PBS and fixed. The fixed cells were subjected to Alizarin Red S staining^17^ (EMD Millipore, TMS-008-C) according to the manufacturer’s protocol.

### siRNA-mediated gene silencing

CD74 expression was knocked down using a specific small interfering RNA (siRNA; Santa Cruz Biotechnology, Catalog #SC-35023). A non-targeting scrambled siRNA (Catalog #SC-44230) served as the negative control. Transfection medium (Catalog #SC-36868) and transfection reagent (Catalog #SC-29528) were purchased from Santa Cruz Biotechnology and used according to the manufacturer’s instructions with minor modifications. Briefly, cells were seeded in antibiotic-free growth medium 24 h prior to transfection. Cells were then transfected with either CD74 siRNA or control siRNA in transfection medium using the transfection reagent and incubated for 6 h at 37 °C in a humidified atmosphere containing 5% CO_2_. Following transfection, the medium was replaced with standard growth medium, and cells were cultured for an additional 24 h before sample collection or induction of osteogenic or chondrogenic differentiation.

### Plasmid-mediated overexpression of CD74

For CD74 overexpression studies, human bone marrow–derived mesenchymal stromal cells (hBMSCs) were transiently transfected with pcDNA3.1 expression vector encoding either CD74 transcript variant 1 (V1; GenScript, Catalog #OHu19995) or transcript variant 3 (V3; GenScript, Catalog #OHu20017). Cells transfected with the empty pcDNA3.1 vector served as controls. Transfections were performed using Lipofectamine™ 3000 (Invitrogen, Thermo Fisher Scientific) according to the manufacturer’s protocol. Following transfection, cells were maintained under standard culture conditions and subsequently harvested for downstream analyses or subjected to osteogenic or chondrogenic differentiation assays.

### Osteogenic differentiation of hBMSCs

Human bone marrow–derived mesenchymal stromal cells (hBMSCs) were induced toward the osteogenic lineage using the MesenCult™ Osteogenic Differentiation Kit (Human; Catalog #05465, STEMCELL Technologies) according to the manufacturer’s instructions. Breifly, hBMSCs were cultured in standard expansion medium until reaching approximately 80%-90% confluence. Cells were then washed once with PBS, and the culture medium was replaced with complete osteogenic differentiation medium. Cultures were maintained at 37 °C in a humidified atmosphere containing 5% CO_2_. The osteogenic medium was replenished every 3 days throughout the differentiation period. osteogenic differentiation was assessed by ALP staining and Alizarin Red S staining after 10 days of induction, and images were acquired using a camera and bright-field microscope. All differentiation experiments were performed using at least 3 biological replicates and repeated independently to confirm reproducibility.

### Chondrogenic differentiation of hBMSCs

Chondrogenic differentiation of hBMSCs was performed using the MesenCult™-ACF Chondrogenic Differentiation Kit (STEMCELL Technologies, Catalog #05455) according to the manufacturer’s protocol. Briefly, hBMSCs were harvested and resuspended in complete chondrogenic medium. 2 × 10^5^ cells were transferred into 15-mL conical tubes and centrifuged at 300× *g* for 5 min to form cell pellets. The pellets were cultured in 0.5 mL of complete chondrogenic differentiation medium at 37 °C in a humidified incubator containing 5% CO_2_ for 21 days. The medium was changed every 2-3 days. At the end of the differentiation period, cell pellets were collected, fixed in 4% paraformaldehyde overnight, embedded in paraffin, and sectioned for histological analysis. Chondrogenic differentiation was assessed by Alcian Blue staining to detect sulfated glycosaminoglycans within the extracellular matrix. The expression of chondrogenic marker COL2A1 was further evaluated by Immunohistochemistry. All experiments were performed in triplicate, and data were obtained from at least 3 independent biological replicates.

### RT-PCR analysis

Total RNA from each sample was isolated using the Aurum Total RNA Mini Kit (Bio-Rad #7326820) according to the manufacturer’s instructions. Isolated RNA was dissolved in RNase-free water, and RNA concentration was determined using a NanoDrop2000 spectrophotometer. After that, the RNA samples were either stored at −80 °C or used immediately. cDNA was prepared using the iScript™ cDNA Synthesis Kit (Bio-Rad #1708891). 1 μg of total RNA was reverse-transcribed in a 20 μL reaction. The final reaction volume was diluted to 100 μL with RNA/DNA-free water. SsoAdvanced™ Universal SYBR® Green Supermix (Bio-Rad #1725271) was used for quantitative PCR (qPCR). A 2-step cycling protocol, consisting of denaturation at 95 °C for 10 s and annealing/extension at 60 °C for 30 s, was performed using an iCycle instrument (Bio-Rad). To normalize cDNA input across samples, 18S rRNA was used as the endogenous control. Each cDNA was analyzed in triplicate.

### Single-cell sample preparation

A preliminary assessment of cell viability and cell count was performed prior to the scheduled experiment. Cryopreserved cells were thawed approximately 5 days before the single-cell sequencing experiment. Cells were prepared according to the General Cell Preparation Protocols on the 10x Genomics website. Briefly, using a wide-bore pipette tip, cells were gently and thoroughly mixed in suspension. Cell concentration was determined using a hemocytometer. A 100 μL cell suspension was prepared at a concentration of 700–1200 cells/μL in 1X PBS-0.04% BSA, kept on ice, and sent to the genome core for processing using the appropriate 10X Genomics Single Cell protocol. The entire sample preparation process was completed within 30 min. Single-cell RNA sequencing was performed on the following cell lines: L8M20 (passages P3 and P5), 23M31 (passages P3 and P5), L32F58 (passage P3), and L11M37 (passage P5). Preliminary analyses indicated a high degree of similarity between the P3 and P5 samples derived from the same donor. Therefore, data from passages P3 and P5 were combined and treated as a single biological sample for downstream analyses when comparing cell lines derived from different donors.

### Single-cell RNA library preparation and sequencing

Sequencing libraries were prepared using the Chromium Single Cell 5′ Kit (10x Genomics, Pleasanton, CA, USA) and sequenced on HiSeq2500 (Illumina, San Diego, CA, USA). Transcripts were mapped using Cell Ranger Pipeline v3 (10x Genomics). Library construction and sequencing were performed at the Genomics Core at LSU Health Shreveport.

Protocol Summary: Four live cell samples were assessed and processed for 10x Genomics single-cell 5′ sequencing with the Chromium Next GEM Single Cell 5′ Reagent Kit v2 (Dual Index, 10x Genomics). GEMs (Gel Bead-in Emulsions) were generated using a Chromium Controller (10x Genomics). RNA in GEMs was reverse transcribed and barcoded, and cDNA was recovered. cDNA was assessed using the Agilent TapeStation D5000 High Sensitivity Kit (Agilent Technologies). 5′ gene expression (GEX) libraries were prepared by fragmentation, end repair, and size selection of cDNA, followed by adaptor ligation and cleanup. Dual indexes were attached via PCR amplification. Completed libraries were purified and assessed for quality using the Agilent TapeStation D5000 High Sensitivity Kit. Libraries were quantitated by qPCR (NEBNext Library Quant Kit for Illumina, Bio-rad CFX96 Opus Real-Time PCR). Libraries were normalized to 1.5 pM and pooled. A 1.5 pM PhiX control library was spiked in as an internal control. The pooled library was denatured and diluted to approximately 300 pM. Paired-end 26 x 90 base pair sequencing was performed on an Illumina NovaSeq 6000 platform.

Analysis Summary: Primary analysis, including base calling and quality scoring, was performed onboard with the Illumina NovaSeq 6000 (NovaSeq Control Software v1.8; RTA v3). Samples were demultiplexed, and FASTQ files were generated for downstream analysis.

### Bioinformatics analysis

Single-cell RNA sequencing analysis was performed using the Cell Ranger pipeline^18^ (v7.1.0) with the GRCh38-2020-A reference transcriptome. The advanced analysis was performed using the Seurat R package^19^ v5.1.0 for quality control filtering, normalization, dimensionality reduction, differential expression gene (DEG) analysis, and integration.^20^ Cells with mitochondrial RNA content >5% were excluded, and cells with fewer than 200 or more than 6000 detected genes were removed to retain the major cell population and exclude potential doublets. Visualizations were generated using the Seurat and ggplot2 R packages (v3.5.1.) Pathway analysis was performed using Qiagen Ingenuity Pathway Analysis (IPA). Genes with a fold change ≥1.8 (upregulated) or ≤0.6 (downregulated) and *P* < .05 were considered significantly differentially expressed.

To assess heterogeneity within each stem-cell sample, we analyzed Cell Ranger–derived UMAP coordinates and graph-based clusters separately for each individual. Marker genes for each local cluster were extracted from Cell Ranger differential expression outputs using adjusted *P* < .05 and log2FC > 0.25. For functional annotation, up to the top 150 marker genes ranked by log2FC were evaluated using Gene Ontology (GO) Biological Process enrichment. Local clusters were assigned to broad functional programs based primarily on marker-gene patterns, with GO enrichment used as supportive evidence where available. Clusters with limited marker support or non-significant enrichment were classified as unresolved. Because each sample was embedded and clustered independently, local cluster labels were treated as sample-specific rather than directly matched across individuals. log2FC > 0.25 was used as a permissive threshold for functional annotation rather than as a criterion for defining strong differentially expressed genes. This threshold retained moderately enriched marker genes for downstream GO and marker-pattern interpretation, while adjusted *P* < .05 was maintained to control statistical significance.

To evaluate whether the analyzed stem-cell compartment showed a BM-MSC-like RNA marker profile, we examined selected marker genes commonly used for MSC characterization. These genes were organized into 3 predefined categories: MSC-associated positive markers, trophic/angiogenic genes, and hematopoietic/immune negative markers. CD marker names were mapped to their corresponding gene symbols, including CD29/ITGB1, CD71/TFRC, CD73/NT5E, CD90/THY1, CD105/ENG, CD106/VCAM1, CD124/IL4R, CD146/MCAM, and CD45/PTPRC. VEGF was represented by VEGFA. For each individual sample, we calculated the percentage of cells with detectable RNA expression and the average log-normalized expression for each selected gene using the filtered feature-barcode matrices. Marker groups were then summarized to assess the overall MSC-like marker profile and the presence or absence of hematopoietic/immune marker signal. These analyses were used as supportive RNA-level marker-profile checks rather than definitive protein-level validation.

## Results

### Characterization of MSCs isolated from hBMSCs

**Figure 1.**
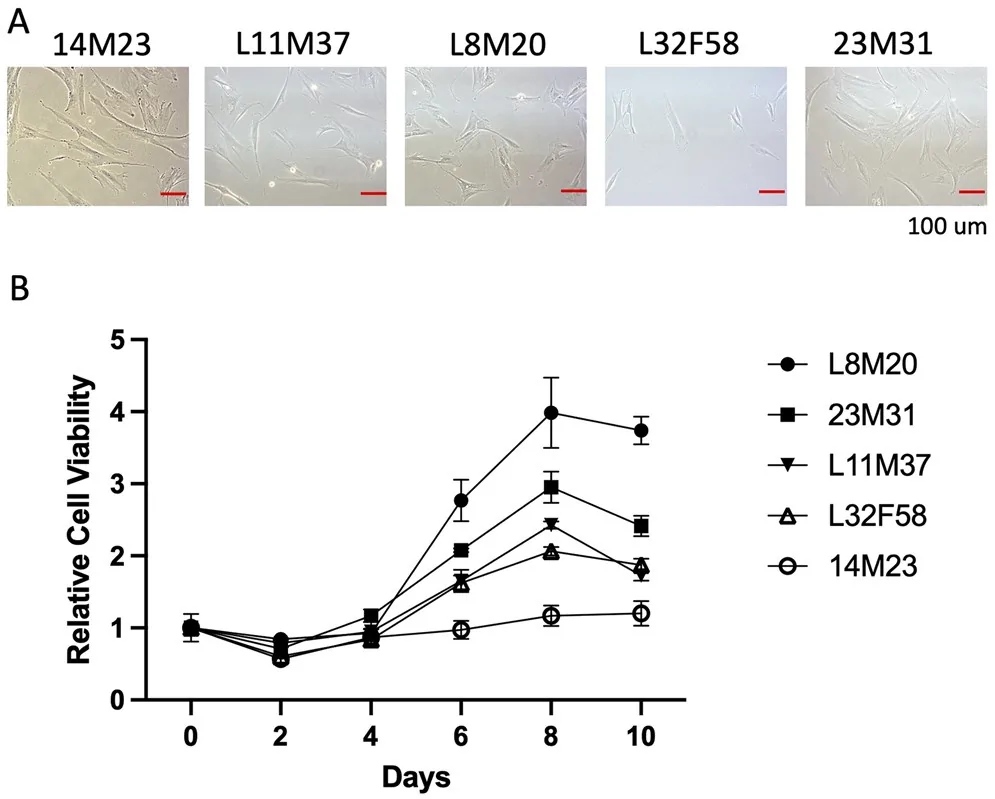
Morphology and growth characteristics of BMSCs derived from different donors. (A) Morphology of monolayer cells isolated from 5 human BMSC lines (14M23, L11M37, L8M20, L32F58, and 23M31) cultured in phenol red-free α-MEM medium with 10% heat-inactivated FBS. (B) Growth rate of BMSCs derived from different individuals assessed by cell counting and CCK8 assay; all data normalized with the day 0 cell number or OD410 determination.

MSCs isolated from human bone marrow were cultured in phenol red-free α-MEM medium containing 10% Fetal Bovine Serum–Heat Inactivated (FBS-HI), and antibiotics (100 U/mL penicillin and 100 μg/mL streptomycin, Invitrogen). The morphology of the BMSCs from 5 different patients (Figure 1A) was observed under a light microscope. The majority of cells displayed a small, spindle-shaped morphology, with a few large, flattened cells present. Cell growth rates were measured for up to 10 days in culture. Our data demonstrated substantial variation in proliferation among samples. For example, MSCs from patient L8M20 exhibited the highest growth rate at day 8, whereas cells from patient 14M23 showed the lowest growth rate at the same time point (Figure 1B). Importantly, CD34 and CD45 (PTPRC), 2 typical negative markers of MSCs (Figure S1A, see online supplementary material for a color version of this figure), were not detected in any tested samples. In contrast, classical positive MSC markers were highly expressed, including CD44, CD73 (NT5E), and CD105 (ENG) (Figure S1A, see online supplementary material for a color version of this figure). Additionally, THY1, also known as CD90 and a member of the immunoglobulin superfamily,^15^ was highly expressed in all MSC samples.

### Osteogenic differentiation capacity of BMSCs from different individuals

**Figure 2.**
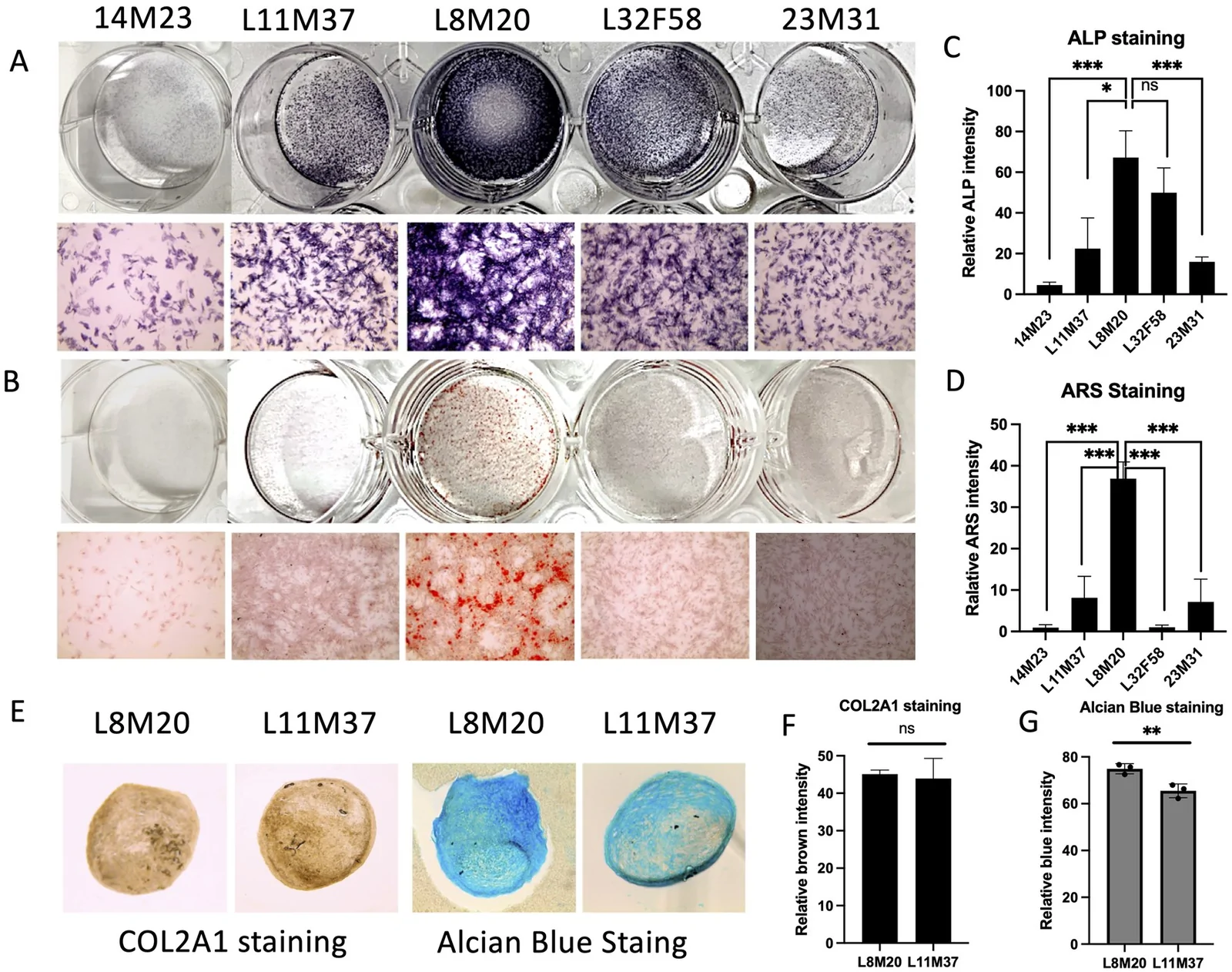
Osteogenic and chondrogenic differentiation potential of BMSCs. (A) Representative images of alkaline phosphatase (ALP) staining after osteogenic induction in 5 BMSC lines (14M23, L11M37, L8M20, L32F58, and 23M31). (B) Representative images of Alizarin Red S (ARS) staining showing calcium mineral deposition following osteogenic differentiation. (C, D) Quantification of ALP and ARS staining intensity using ImageJ. * indicate statistical difference with *P* <0.05, ** indicate statistical difference with *P* <0.01, *** indicate statistical difference with *P* <0.001. (E) Representative images of type II collagen (COL2A1) immunostaining and Alcian Blue staining of chondrogenic pellets generated under three-dimensional (3D) chondrogenic culture conditions from L8M20 and L11M37 cells. (F, G) Quantification of COL2A1 and Alcian Blue staining intensity.

The osteogenic differentiation potential of BMSCs was assessed by ALP staining and Alizarin Red S (ARS) staining. All cell lines exhibited positive ALP activity (Figure 2A) and ARS staining (Figure 2B), with L8M20 demonstrating the strongest osteogenic activity. Quantitative analyses of ALP activity and ARS staining, performed using ImageJ, are presented in Figure 2C and D. Because chondrogenic differentiation is directly relevant to musculoskeletal tissue repair, we also evaluated the chondrogenic potential of BMSCs by inducing pellet formation under three-dimensional (3D) chondrogenic culture conditions. Representative Alcian Blue staining and type II collagen (Col II) staining images demonstrated the cells’ capacity for chondrogenic differentiation (Figure 2E-G).

### The global GEX in high- and low-osteogenic differentiation capacity BMSCs

**Figure 3.**
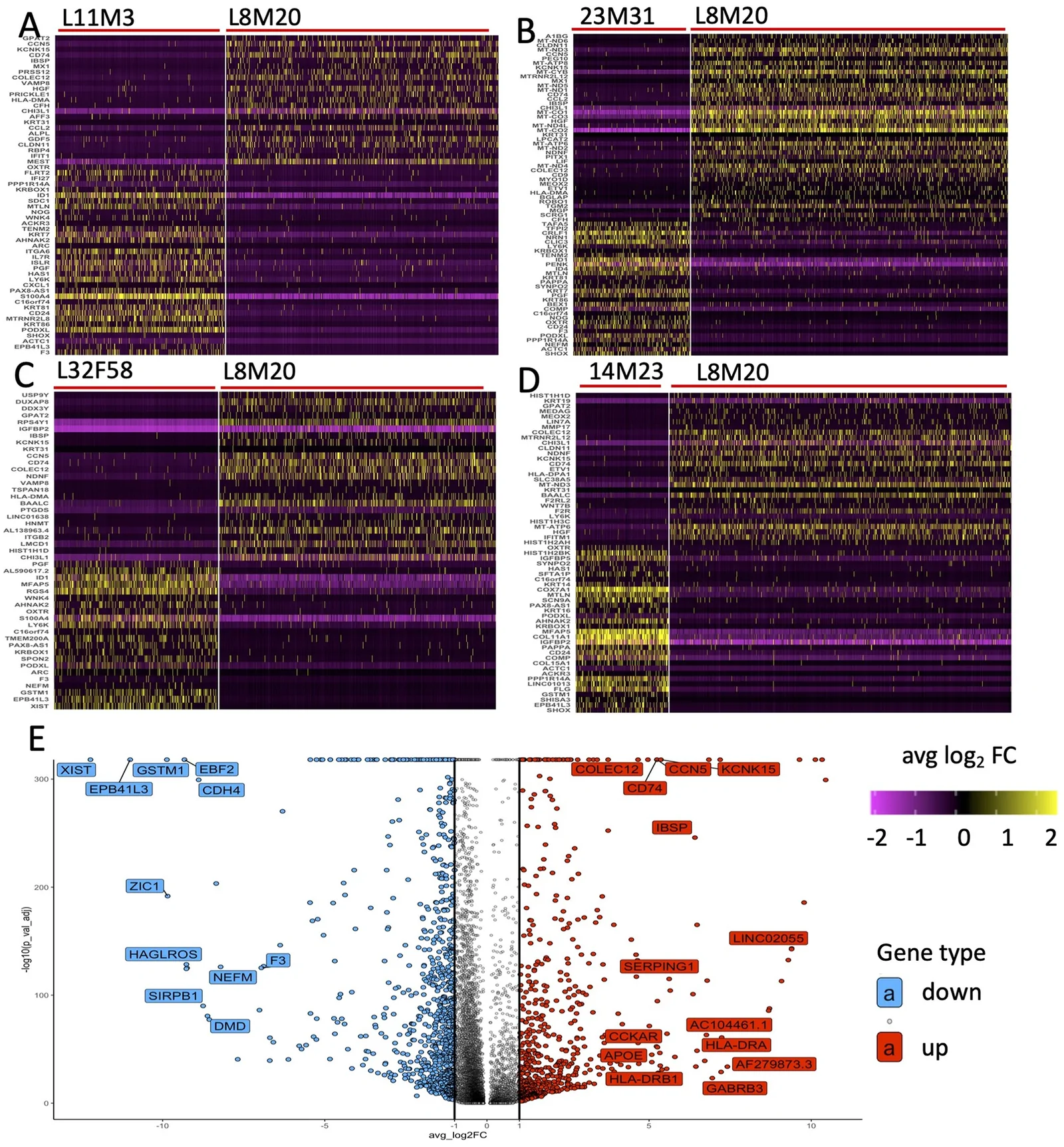
Differential gene expression between L8M20 and other BMSC samples. (A-D) Top significantly changed genes in L8M20 compared to L11M37 (A), 23M31 (B), L32F58 (C), and 14M23 (D). (E) Volcano plot showing differentially expressed genes in L8M20 vs other cells. Red dots represent genes upregulated in L8M20; blue dots indicate genes upregulated in other cells. Y-axis: −log10 *P*-value; X-axis: log2 fold change.

To evaluate global GEX differences among BMSCs derived from different individuals, we compared GEX profiles between the samples with high and low osteogenic differentiation capacities. The top significantly differentially expressed genes were selected for visualization in a heatmap (Figure 3). Transcriptome analysis revealed that in L8M20, 168 genes were upregulated and 368 genes were downregulated by more than 2-fold compared with the other 4 cell lines. The top 10 upregulated genes in L8M20 were KCNK15, CD74, CCN5, IBSP, COLEC12, CHI3L1, HLA-DMA, NDNF, HGF, and GPAT2. In contrast, the top 10 downregulated genes were SHOX, EPB41L3, ACTC1, F3, PODXL, CD24, PPP1R14A, C16orf74, OXTR, and KRBOX1.

### UMAP feature plots of BMSCs from different individuals

**Figure 4.**
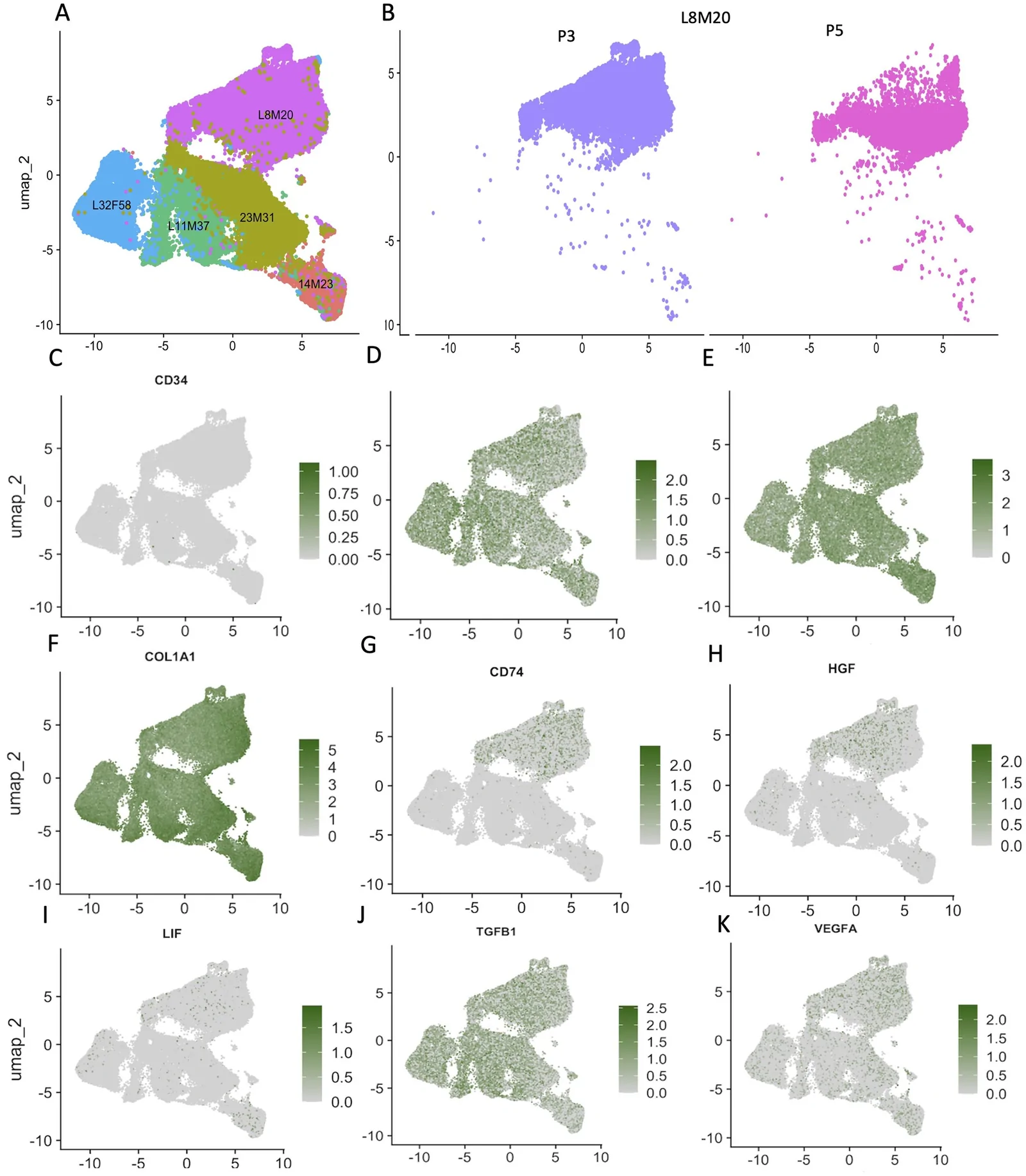
UMAP feature plots displaying gene expression patterns across different BMSC samples. Green dots represent individual cells expressing the corresponding gene. (A) UMAP plot showing cell distribution by sample. (B) Reproducibility of single-cell 5′ sequencing results in P3 and P5 passages. (C-E) CD44, CD34, and THY1expression patterns, markers of stem cell. (F-K) Selective genes expression of osteogenic/chondrogenic marker genes COL1A1, CD74, HGF, LIF, TGF-β, and VEGFA.

The global feature map of BMSCs (Figure 4A) illustrates the transcriptomic landscape of individual cells derived from different donors. The UMAP visualization reveals non-uniform GEX distributions within each group, indicating notable transcriptional heterogeneity. Figure 4C-E displays the distribution of selected marker genes associated with osteogenic and chondrogenic differentiation potential across the UMAP clusters.

CD34, CD44, and CD90 as markers of MSC identity (positive or negative expression) showed expression patterns consistent with expectations (Figure 4C-E). The compact clustering patterns within each group suggest that monolayer cells isolated from bone marrow contain a high proportion of cells with shared stem cell characteristics. COL1A1, CD74, HGF, TGF-β, LIF, and VEGFA were selected as osteogenic and chondrogenic predictive marker genes (Figure 4F-K).

Among these genes, CD74 was the most prominent in the distribution maps (Figure S1A and B, see online supplementary material for a color version of this figure), showing significantly higher expression in L8M20 compared with all other groups. This sample also demonstrated the strongest osteogenic differentiation capacity. Additionally, BMSCs at passages 3 and 5 exhibited high transcriptomic similarity, as demonstrated by the overlapping distributions of L8M20 P3 and P5 (Figure 4B), as well as 23M31 P3 and P5. In addition to identifying CD74 as a differentially expressed gene, the single-cell RNA sequencing analysis provided insight into the overall cellular composition of donor-derived BMSCs. Consistent with the ISCT criteria for MSC characterization, cells from all donors expressed classical MSC markers, including CD44, CD73, CD90 (THY1), and CD105, while lacking expression of hematopoietic markers. Although the donor-derived populations exhibited substantial overlap in UMAP space, distinct differences in GEX patterns were observed among samples. Notably, CD74-positive cells were markedly enriched in L8M20, the donor displaying the highest osteogenic differentiation capacity. Furthermore, the close overlap between passage 3 and passage 5 samples suggests that the transcriptional profile of these cells remains relatively stable during short-term in vitro expansion, supporting the reproducibility of the observed donor-specific signatures.

### Pathway analysis of different individual-derived BMSCs by QIAGEN ingenuity pathway analysis

**Figure 5.**
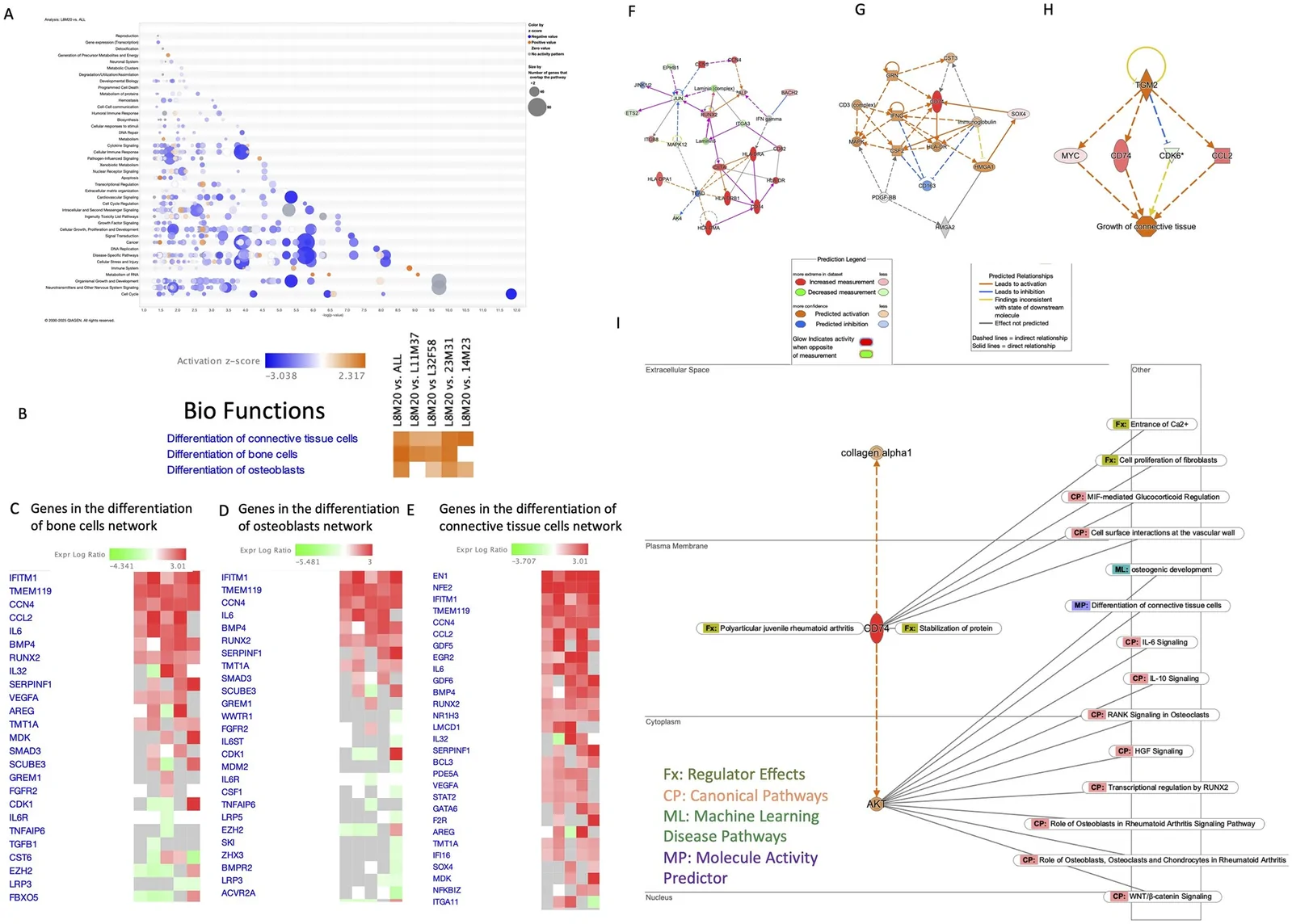
Pathway and network analysis comparing L8M20 to other BMSC samples. (A) Bubble chart illustrating pathway enrichment; Y-axis: pathway names; X-axis: −log10 *P*-value; bubble size: number of enriched genes; color: *Z*-score (orange = upregulated; blue = downregulated). (B) Heatmap of pathways related to differentiation of connective tissue, bone cells, and osteoblasts. (C-E) Differential gene expressions in pathways shown in (B). (F-H) Merged networks involving CD74. (I) Regulatory network analysis showing genes affected by CD74 during osteogenesis.

The majority of canonical pathways were predicted to be inhibited in L8M20 compared with other BMSCs, particularly pathways related to cell cycle progression and DNA replication, which exhibited high absolute z-scores (Figure 5A and Figures S2 and S3, see online supplementary material for a color version of these figures). The 14M23 cell line demonstrated the slowest proliferation rate among all samples and showed marked suppression of cell cycle pathways, consistent with its growth characteristics. The top 5 significant canonical pathways were cell cycle checkpoints, axonal guidance signaling, mitochondrial RNA degradation, interferon alpha/beta signaling, and the pulmonary fibrosis idiopathic signaling pathway. The upstream regulators ERBB2, TGFB1, and PPARD were predicted to be inhibited in L8M20. In the causal network, TGM2, SRC family kinases, and TIMP1 were predicted to be inhibited, while BRCA1 was predicted to be activated. Alkaline phosphatase, a marker commonly used to assess osteogenic activity, was identified in the IPA Top Tox Functions analysis as significantly increased in L8M20, consistent with our ALP staining results.

Furthermore, pathways associated with “differentiation of bone cells,” “differentiation of osteoblasts,” and “differentiation of connective tissue cells” were significantly increased in L8M20 compared with other samples (Figure 5B-E). However, CD74 did not appear in the analysis of any of these pathways.

To further investigate the potential role of CD74 in osteogenic differentiation, we examined the interaction networks in which CD74 is involved. CD74 participates in several canonical pathways, including antigen presentation, macrophage migration inhibitory factor (MIF)-mediated glucocorticoid regulation, cell surface interactions at the vascular wall, MHC class II antigen presentation, and MIF regulation of innate immunity. Analysis of the merged CD74 interaction network (Figure 5F-H) revealed several potential connections between CD74 and bone differentiation processes^21^ (Figure 5I).

### Individual-level UMAP analyses for each stem-cell sample

Individual-level UMAP analysis showed that stem cells from each sample formed multiple local graph-based clusters, supporting intra-sample transcriptional heterogeneity (Figure S4A, see online supplementary material for a color version of this figure). Marker-based annotation, supported where applicable by GO Biological Process enrichment, organized these local clusters into recurrent functional programs, including cycling/proliferative, ECM/adhesion-associated, immune-response/chemotaxis-associated, mitochondrial-high, and developmental/lineage-priming programs (Figure S4B and C, see online supplementary material for a color version of this figure). Clusters with limited marker or enrichment support were conservatively retained as unresolved. Together, these analyses show that stem-cell heterogeneity was detectable within individual samples and was not solely driven by between-individual separation in the integrated analysis.

### BM-MSC-related RNA marker-profile analysis across the 5 individual samples

The selected MSC-associated surface-marker transcripts were broadly detected across the 5 individual stem-cell samples, particularly ITGB1/CD29, CD44, THY1/CD90, ENG/CD105, and NT5E/CD73. In contrast, hematopoietic/immune negative markers, including CD14, CD34, PTPRC/CD45, and HLA-DRA, showed minimal detection. The trophic/angiogenic genes, including ANGPT-family genes, VEGFA, FGF2, HGF, and IGF1, were detected at lower and more variable levels across individuals (Figure S5, see online supplementary material for a color version of this figure). Together, these findings support an MSC-like RNA-level marker profile of the analyzed stem-cell compartment.

### CD74 plays critical role in osteogenic differentiation

**Figure 6.**
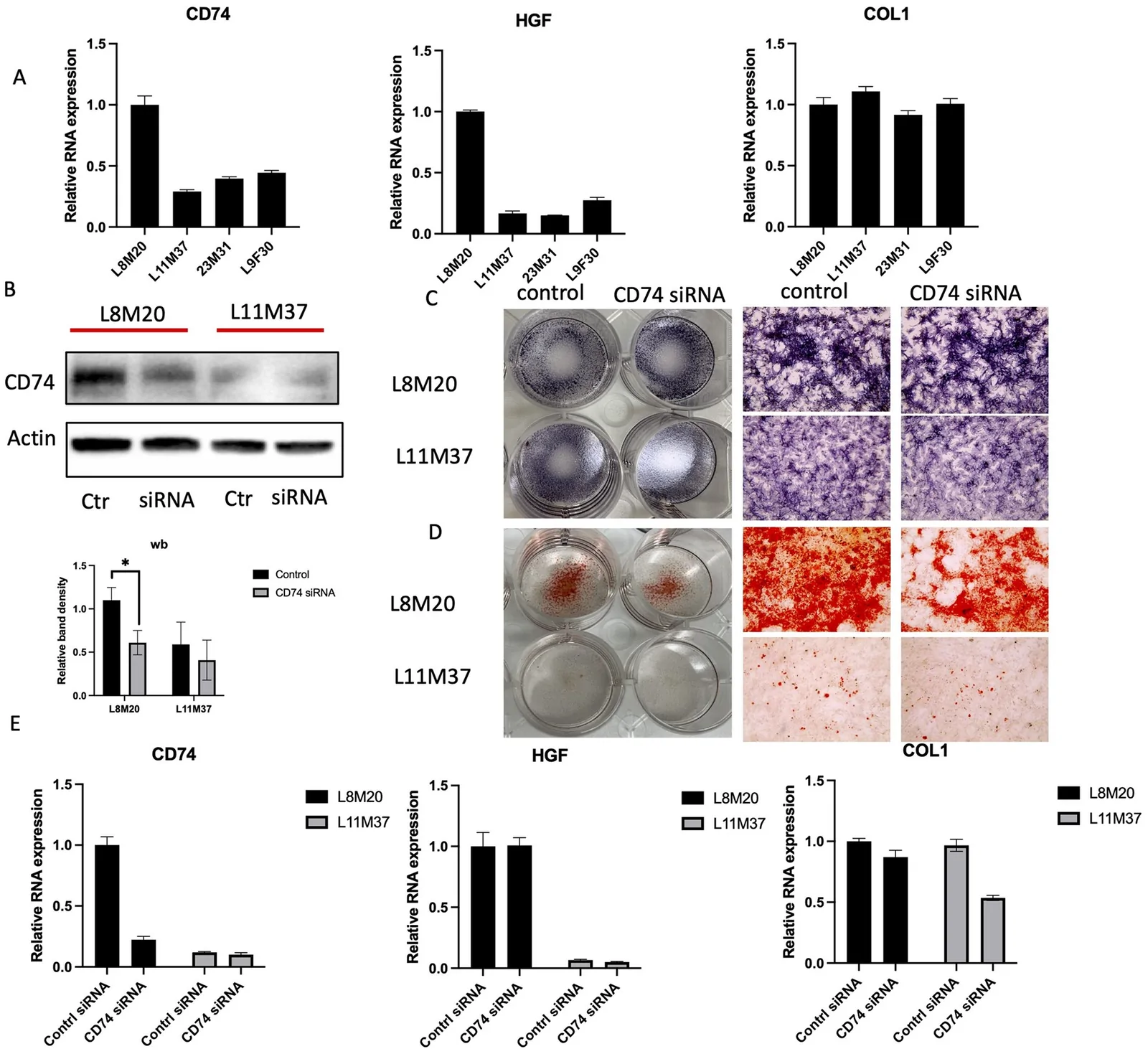
Knockdown of CD74 reduces osteogenic activity in BMSC. (A) qPCR validation of CD74 and HGF expression: L8M20 cells showed higher expression than other lines; COL1A1 expression was similar across samples. (B) Western blot confirming CD74 knockdown in L8M20; no significant change in L11M37. * indicate statistical difference with *P* <0.05. (C) ALP activity in L8M20 and L11M37 following CD74 knockdown. (D) Osteoblast activity assessed by ARS staining in L8M20 and L11M37 after CD74 knockdown. (E) Gene expression changes following CD74 knockdown in L8M20 (high CD74) and L11M37 (low CD74).

To verify the role of CD74 in osteogenic differentiation, we performed CD74 KD using CD74-specific siRNA. CD74 expression was reduced at both the mRNA and protein levels in L8M20 following siRNA treatment (Figure 6A and B), whereas no significant changes were observed in L11M37. Alkaline phosphatase staining did not show significant differences between CD74 KD and control cells in either L8M20 or L11M37 (Figure 6C). In contrast, calcium-rich deposits were markedly reduced in L8M20 following CD74 KD, whereas no significant changes were observed in L11M37 (Figure 6D). Interestingly, COL1A1 expression was slightly decreased in CD74 KD cells, while HGF expression remained unchanged (Figure 6E).

## Discussion

Human bone marrow is a soft, spongy tissue at the center of bones that produces blood cells and contains supporting cells such as fibroblasts, macrophages, adipocytes, osteoblasts, osteoclasts, and endothelial cells, as well as MSCs. In clinic, bone marrow aspirate concentrate (BMAC) is widely used to enhance skeletal tissue repair as a concentrated solution containing stem cells, anti-inflammatory cytokines, and growth factors that promote cellular growth and tissue repair. Although transplantation of BMAC for tissue repair shows promising outcomes, significant concerns remain, including variable outcomes after injection of BMAC and the unknown underlying mechanisms that drive damaged tissue regeneration. Previous research has identified BMSCs as the major cell source in BMAC for skeletal tissue repair,^22^ but their exact contribution is still unknown. Additionally, although BMSCs are widely used in skeletal tissue repair, their molecular signature remains poorly defined. Since BMSCs represent a highly heterogeneous population that creates challenges for routine clinical applications,^23^ identification of factors responsible for BMAC repair ability is critical for predicting long-term outcomes.

Recent advances in single-cell transcriptomic technologies have provided important insight into the cellular heterogeneity of BMSCs. Although MSCs are usually defined by the expression of surface markers such as CD73, CD90, and CD105, increasing evidence suggests that these populations contain multiple functionally different subpopulations with different regenerative capacities. Previous studies have identified stromal and mesenchymal subpopulations associated with osteogenic differentiation, tissue regeneration, and support functions. Consistent with these observations, our results demonstrate that donor-derived BMSCs expressed high level of classical MSC markers while exhibiting substantial differences in osteogenic potential and transcriptional profiles. These findings support the concept that conventional MSC markers alone may be insufficient to predict regenerative performance and highlight the value of identifying biomarkers associated with functional capacity.^24–26^ Similar to previously reported single-cell transcriptomic studies that identified osteogenic-prone stromal subpopulations, our findings suggest that donor-specific differences in GEX may contribute to the variability in osteogenic capacity observed among BMSC preparations.^24–26^

It has been established that BMSCs are stromal cells capable of differentiating into osteoblasts, chondroblasts, and adipocytes. They express biomarkers including CD73, CD90, and CD105, and lack expression of CD34, CD45, CD19, or CD79α.^15^ To investigate the difference of BMSCs from different patients, we first isolated BMSCs from 5 different patients and then compared GEX pathways between samples that have high osteogenic activity and samples that have low osteogenic potential. As shown in our data, all BMSCs from 5 patients expressed these typical stem cell markers, but their osteogenic potential varied widely. Since numerous proteins interact within signaling pathways to form complex networks that regulate biological function, we further performed single-cell RNA sequencing to identify genes correlated to this difference. Our data showed that genes involved in differentiation of connective tissue cells, bone cells, and osteoblasts were significantly upregulated in BMSCs from L8M20, which showed the highest osteogenic differentiation potential (Figure 5B-E). Interestingly, global pathway comparison revealed that some pathways were significantly reduced in BMSCs from L8M20 when compared to the other samples that have lower osteogenic potential (Figure S2, see online supplementary material for a color version of this figure). Similarly, a stacked bar chart comparing functional and disease-related pathways between BMSCs from L8M20 and BMSCs from other patients (Figure S3, see online supplementary material for a color version of this figure) display similar results that most pathways were inhibited in BMSCs from L8M20. These findings suggest that lower transcriptional activity in some pathways may create a favorable cellular microenvironment for stem cell differentiation toward bone tissue.

Individual-level UMAP analyses of each stem-cell sample demonstrated that stem-cell heterogeneity was detectable within individual samples and was not solely attributable to between-donor variation observed in the integrated analysis (Figure S4, see online supplementary material for a color version of this figure). Furthermore, BM-MSC marker expression profiling across the 5 individual stem-cell samples confirmed that all samples exhibited established stem-cell characteristics based on currently recognized BM-MSC markers (Figure S5, see online supplementary material for a color version of this figure). However, differences in osteogenic differentiation capacity among these samples suggest that the existing marker set may not be sufficient to accurately predict stemness or functional potential. Specifically, in the context of osteogenic differentiation, additional biomarkers may exist that could improve the assessment of stem-cell quality and facilitate more effective clinical selection of BM-MSCs. Using IPA network analysis, we further identified CD74 as a potential key marker in osteogenic differentiation. CD74 is a type II transmembrane protein, also known as the MHC class II-associated invariant chain.^24^

Although CD74 has been classically characterized as an invariant chain involved in MHC class II antigen presentation and immune regulation, accumulating evidence indicates that its biological functions extend well beyond the immune system. Recent studies have shown that CD74 serves as a cell-surface receptor for macrophage MIF, a pleiotropic cytokine secreted by monocytes/macrophages and activated T lymphocytes. The interaction between CD74 and MIF activates multiple downstream signaling pathways, including ERK, AKT, and NF-κB, thereby influencing diverse cellular processes such as proliferation, survival, differentiation, apoptosis, and tissue repair. Notably, these effects appear to be highly cell type-dependent. Given the established roles of MIF–CD74 signaling in regenerative and reparative responses, it is plausible that CD74 contributes to stem cell regulation and tissue regeneration through mechanisms that are distinct from its traditional immunological functions. These findings support an emerging view of CD74 as a multifunctional regulator involved in both immune and non-immune biological processes.^27–31^ For example, in Hematopoietic stem and progenitor cells (HSPCs), CD74 interacts with the transcription factors RUNX and NF-κB and binds to proximal and distal regulatory sites enriched for genes involved in apoptosis, immune response, and cell migration.^32^ Previous studies have also shown that soluble MIF binding with CD74 impairs human bone marrow MSC healing function by blocking the migration of MSCs into the injured sites^33^; In contrast, CD74 stimulation with anti-CD74 antibody leads to an induction of a signaling cascade resulting in NF-kappaB activation, entry of the stimulated cells into the S phase, elevation of DNA synthesis, and cell division suggesting that surface CD74 may function as a cell survival receptor.^34^

Although the different biological activities of CD74 in multiple cell types were reported, the potential role of CD74 in MSC osteogenic differentiation has not yet been investigated. Our data generated from human bone marrow MSCs suggested that CD74 may also influence osteogenesis through regulation of downstream proteins such as collagen type I alpha 1 and AKT, as well as pathways including calcium signaling. More importantly, when CD74 was knocked down in BMSCs with high osteogenic capacity, we observed a significant decrease of the mineralized nodule formation in culture indicating high level of CD74 is required for enhanced BMSC osteogenic potential. As a cell surface receptor, CD74 interacts with CD1D, COPG2, MHC II, COPG1, YWHAZ, NCAPD3, CXCR2, CCR1, HLA-DR, and HLA-DQB1 that are associated with mediation of immunoresponse; furthermore, it regulates genes related to triacylglycerol metabolism, including CXCL8, NFkB, p38 MAPK, IL6, SYK, IL2, IgM, IgG2b, and IgG1. In contrast, our data also showed that CD74 can be regulated by several inflammation factors, such as TNF, NFkB, IL4, IL1B, IFN beta, IL15, further confirms that CD74 is involved in immune regulation, intracellular protein transport, macrophage cytokine production, and cytokine-mediated signaling pathways that play critical role in BMAC augmented tissue repair.

Our analysis revealed multiple interactions between CD74 and genes involved in extracellular matrix regulation and bone formation. Combined with the reduction in mineralization observed following CD74 KD, these findings support a functional role for CD74 in regulating osteogenic differentiation.^21^^,^^31^ Taken together, our findings suggest that CD74 may provide information beyond that offered by traditional MSC markers. While classical MSC markers were broadly expressed across all donor-derived BMSC populations, CD74 expression was enriched in the donor exhibiting the highest osteogenic capacity and was functionally associated with mineralization following KD studies. These observations support the potential use of CD74 as a predictive biomarker for identifying osteogenically competent BMSC populations and selecting donor-derived cells with high regenerative potential.

Several limitations of this study should be acknowledged. First, the number of donor-derived BMSC samples included in the single-cell RNA sequencing analysis was relatively small, which limited our ability to perform subgroup analyses based on donor characteristics such as age and sex. Second, although BMSCs possess multilineage differentiation potential, our study focused primarily on osteogenic differentiation and included chondrogenic evaluation because of its direct relevance to skeletal tissue regeneration. Adipogenic differentiation was not assessed, therefore, the relationship between CD74 expression and adipogenic lineage commitment remains unclear. Future studies incorporating larger donor cohorts and comprehensive multilineage differentiation analyses will further clarify the role of CD74 in regulating BMSC function and therapeutic potential.

## Conclusion

In this study, we functionally validated the role of CD74 in osteogenic differentiation through KD experiments in BMSCs with high osteogenic potential. In addition, network analysis identified several candidate downstream regulators of CD74, including KCNK15, COLEC12, and PODXL, highlighting potential signaling pathways involved in osteogenesis. Future studies are warranted to characterize the functions of these genes and further elucidate the molecular mechanisms through which CD74 regulates osteogenic differentiation. Collectively, our findings establish CD74 as a novel marker associated with enhanced osteogenic potential in BMSCs. The preferential expression of CD74 in highly osteogenic donor-derived BMSC populations, together with its functional contribution to mineralization, suggests that CD74 may serve as a useful biomarker for identifying BMSC populations with superior bone-forming capacity. Furthermore, CD74-high BMSCs may represent a regenerative subpopulation that contributes to the therapeutic efficacy of BMAC in skeletal tissue repair and regeneration.

## Supplementary Material

szag082_Supplementary_Data

## Data Availability

The datasets generated and/or analyzed in this study are not publicly available due to patient privacy restrictions but are available from the corresponding author upon formal request for research purposes. A confidentiality agreement for data protection regulations and ethical guidelines is required. All requests must include a justification for data use.
