# Supplementary material for: Single‐cell transcriptomics reveals critical role of CD74 in bone marrow–derived mesenchymal stem cells
Source: Stem Cells Transl Med. 2026 Sep 18;15(10):szag082. doi: 10.1093/stcltm/szag082 (PMC13588712; doi:10.1093/stcltm/szag082)
Supplement: Supplementary file 1 [file szag082_supplementary_data.docx]

**Supplementary Information**

Single‐Cell Transcriptomics Reveals Critical Role of CD74 in Bone Marrow–Derived Mesenchymal Stem Cells

Running head of the title: CD74 Regulates Osteogenic Capacity in BMSCs

Qinqin Xu^1,2^, Carson Curtis^1^, Jiahao Zhang^2^, Arjun Verma^1^, Aaron J Sheppard ^1^, Jeffrey Deng^3^, Hicham Drissi^4^, Shane Barton^1^, Patrick Massey^1^, Yufeng Dong^1*^

^1^ Department of Orthopedic Surgery, LSU Health-Shreveport, LA 71130 USA

^2^ Department of Neurology, LSU Health-Shreveport, LA 71130 USA

^3^ Dartmouth Geisel School of Medicine, Hanover, NH 03755 USA

^4^ Department of Orthopedic Surgery, Emory University School of Medicine, Atlanta, GA. 30329 USA

Author contributions

Q.X.: Conception and design, Collection and assembly of data, Data analysis and interpretation, Manuscript writing, review and editing; C.C.: Collection and assembly of data, Data analysis, Manuscript writing, review and editing; J.Z.: Data analysis and interpretation, Manuscript review and editing; A.V.: Provision of study material or patients; A. J. S.: Methodology, Provision of study material or patients; J.D.: Data analysis, Manuscript writing, review and editing; H.D.: Data analysis, review and editing; S.B.: Conception, Administrative support; P. M.: Provision of study material or patients, Administrative support; Y. D.: Conception and design, Financial support, Administrative support, Manuscript review and editing

Original Research

Keywords

Bone marrow-derived mesenchymal stromal cells (BMSCs), Single-Cell RNAseq, Osteogenesis, CD74

^*^Corresponding author: Yufeng Dong, Email: [yufeng.dong@lsuhs.edu](mailto:yufeng.dong@lsuhs.edu). Phone: 318-675-3252.

Mailing address: Department of Orthopedic Surgery, 1501 Kings Highway, Shreveport, LA 71130 USA,

Funding

This work was supported in part by the following grants: a grant from the National Institute of Arthritis and Musculoskeletal and Skin Diseases of the National Institutes of Health (Grant No. R01AR073277-01A1) to Y.D.

Figure S1

Gene expression panels of potential stem cell osteogenic(A) /chondrogenic (B) markers

Figure S2

Heatmap showing canonical pathway comparation between L8M20 and other BMSCs.

Figure S3

Stacked bar chart of function and disease-related pathways comparation between L8M20 and other BMSCs. X-axis: pathway names; Y-axis: proportion of up/downregulated genes; numbers above bars indicate total gene counts.

Figure S4

Individual-level analysis of stem-cell heterogeneity. (A) UMAP visualization of local graph-based clusters within each individual stem-cell sample. Each dot represents one cell, and colors indicate graph-based clusters identified within the corresponding sample. (B) UMAP visualization of the same cells colored by local functional programs. Functional programs were assigned at the local-cluster level using cluster marker genes and Gene Ontology Biological Process enrichment. (C) Dot plot showing the proportion of cells assigned to each local functional program in each individual sample. Dot size indicates the percentage of cells. In each UMAP panel, n denotes the number of analyzed cells, and k denotes the number of local graph-based clusters identified within that individual sample. Because UMAP embeddings and graph-based clusters were generated independently for each sample, local cluster numbers are sample-specific and were not treated as directly matched clusters across individuals. Functional program labels provide biological context for local clusters; clusters with limited marker or GO support were classified as unresolved.

Figure S5

BMSC-related RNA marker-profile check across individual stem-cell samples. (A) Profile-level summary of selected marker groups across individual samples. Values indicate the mean percentage of cells with detectable RNA expression across genes within each marker group. These marker groups are independent gene-set summaries and are not compositional proportions; therefore, values are not expected to sum to 100%. (B) Dot plot showing RNA-level expression of selected BMSC-related marker genes across individual stem-cell samples. Dot size indicates the percentage of cells with detectable expression, and color intensity indicates average log-normalized expression. CD marker names were mapped to their corresponding gene symbols, including CD29/ITGB1, CD73/NT5E, CD90/THY1, CD105/ENG, CD106/VCAM1, CD146/MCAM, and CD45/PTPRC. VEGF was represented by VEGFA. These analyses were used as supportive RNA-level marker-profile checks and were not intended as definitive protein-level MSC validation.
